# Circulating Tumor Cells Predict Response of Neoadjuvant Chemotherapy in Patients with Bladder Cancer: A Preliminary Study

**DOI:** 10.3390/diagnostics13061032

**Published:** 2023-03-08

**Authors:** Yu-Cing Jhuo, Tai-Lung Cha, Chien-Chang Kao, Yi-Ta Tsai, Sheng-Tang Wu, En Meng, Chih-Wei Tsao, Chin-Li Chen, Hui-Kung Ting, Guang-Huan Sun, Dah-Shyong Yu, Sun-Yran Chang, Ming-Hsin Yang

**Affiliations:** 1 Division of Urology, Department of Surgery, Tri-Service General Hospital, National Defense Medical Center, Taipei 114, Taiwan; 2 Graduate School of Medical Sciences, National Defense Medical Center, Taipei 114, Taiwan

**Keywords:** circulating tumor cells, muscle-invasive bladder cancer, neoadjuvant chemotherapy, radical cystectomy, urothelial carcinoma

## Abstract

This study aimed to explore the existence of circulating tumor cells (CTCs) in patients with muscle-invasive bladder cancer (MIBC) and their predictive potential for response to neoadjuvant chemotherapy (NAC). From 33 blood samples of MIBC patients, CTCs were isolated by cell surface markers and enriched by the IsoFlux™ device, followed by morphological and immunofluorescent identification. CTCs were detected at baseline in all samples. Immunofluorescence confirmed the tumor origin. MIBC patients were stratified by NAC response into the disease control (DC) and progressive disease (PD) groups. In the DC group, the number of CTCs decreased significantly after four courses of NAC (*p* < 0.0001). CTC counts in 7.5 mL after four NAC cycles were highly correlated with postoperative pathological T stage (*p* < 0.0001). Our study demonstrated that CTCs might represent a valuable predictive marker for NAC response in MIBC. CTC detection in MIBC patients could allow early arrangement of radical cystectomy for NAC non-responders to prevent disease progression while receiving the NAC courses.

## 1. Introduction

Bladder cancer is the sixth most common cancer with male predominance in the United States, with 81,180 new cases and 17,100 deaths estimated for 2022 [1]. Muscle-invasive bladder cancer (MIBC) develops metastatic disease within two years of diagnosis and requires radical cystectomy (RC) for treatment [2,3]. Even though RC with pelvic lymph node dissection is performed for patients with MIBC, about half of these patients will eventually develop distant metastatic disease due to initial microscopic metastasis [4,5]. Neoadjuvant chemotherapy (NAC) plays a vital role in MIBC treatment [2]. Various randomized clinical trials have revealed that platinum-based NAC can provide overall survival benefits and decrease the risk of death compared with surgical intervention alone [3,6]. An overall survival benefit of approximately 5% for NAC was found in pure urothelial bladder cancer [7]. However, NAC could affect patients’ quality of life due to side effects and the potentially fatal delay of RC in non-responder patients [8]. NAC might take several months, and monitoring the therapeutic response would be essential during treatment. Furthermore, identifying non-responders is critically important in the setting of NAC in patients with MIBC.

Circulating tumor cells (CTCs) are derived from metastatic lesions or primary tumors and can enter the body through the bloodstream or lymphatics. Through migration, adhesion, and aggregation, CTCs can result in distant metastatic tumor lesions in several cancer types, including urothelial carcinoma (UC) [9,10,11,12,13]. The detection of CTCs in biological fluids in patients with malignancies is thought to be a type of liquid biopsy. CTCs could provide a less invasive technique to access tumor material and would also be available at any time and for sequential sampling. Making use of this non-invasive method, prediction of therapeutic response and monitoring of disease status can be accomplished by liquid biopsy. The detection of CTCs in different cancer types has been widely studied, and progress has been made in research on CTC in UC [14,15,16,17,18]. However, the relationship between CTC numbers in patients with MIBC and therapeutic response to NAC has not been clearly investigated.

To the best of our knowledge, no quantitative parameter has been validated to predict the effect of NAC on MIBC. This study aimed to evaluate the predictive potential of CTC numbers in patients with MIBC who underwent RC following NAC. We used the IsoFlux^TM^ system (Fluxion Biosciences Inc., South San Francisco, CA, USA) to isolate CTCs in 38 blood samples from patients with UC. The CTC platform combines microfluidic channels and immunomagnetic beads targeted to selected antigens on the cancer cell surface to efficiently enrich CTCs. According to changes in tumor stage after treatment, these patients were stratified into disease control (DC) and progressive disease (PD) groups. The disease group was correlated with CTC numbers to explore the predictive potential of CTCs for the therapeutic effect of NAC.

## 2. Materials and Methods

### 2.1. Patient Samples Collection

This study was approved by the institutional ethics committee in Tri-Service General Hospital (TSGH), Taipei, Taiwan (TSGHIRB NO:2-107-05-167). To participate in this study, all patients completed written informed consent. This study included 33 MIBC patients treated by several uro-oncologists at TSGH. From each patient, 7.5 mL of peripheral blood was collected in ethylenediaminetetraacetic acid (EDTA) tubes for processing at three timepoints: before treatment, after four cycles of NAC, and two weeks after radical surgery. All samples were processed within four hours after collection. Further evaluation and CTC analysis were then handled in the National Defense Medical Center (NDMC), Taipei, Taiwan. Five patients with MIBC who did not receive platinum-based chemotherapy before RC were also enrolled in the control group. Furthermore, 7.5 mL of peripheral blood was also collected before RC and two weeks after RC. Blood samples were also processed within 4 h at NDMC.

### 2.2. Immunomagnetic Beads Preparation for CTC Isolation

We modified the CTC enrichment protocol provided by IsoFlux^TM^, which makes replacement of IsoFlux^TM^ beads with CELLection™ Dynabeads (Invitrogen, Waltham, MA, USA) possible. Antibodies are attached to the Dynabeads via a DNA linker. The DNA linker provides a cleavable site to remove the beads from the cells after the enrichment process, which would otherwise not be feasible for the original beads provided by IsoFlux. Further molecular analyses could be performed using separated, ready-for-use cancer cells following immediate access to high-quality, viable cells in a high-density format. CELLection™ Dynabeads^®^ coated with human anti-mouse IgG (Invitrogen) were used for cell isolation. Dynabeads were incubated with EPCAM antibody (Ber-EP4, Abcam, Cambridge, UK; 0.02 µg antibody/µL bead suspension) at room temperature. The beads were stored at 4 °C after being washed with phosphate-buffered saline (PBS) with 0.1% bovine serum albumin (BSA) twice.

### 2.3. Blood Sample Preparation

Red blood cell (RBC) lysis buffer 45 mL containing 0.01 M potassium hydrogen carbonate, 0.1 mM EDTA, and 0.155 M ammonium chloride was used for lysing RBC. The cell pellet was then gently washed in PBS twice and resuspended in Roswell Park Memorial Institute (RPMI) medium containing 1% fetal bovine serum (FBS), 1 mM CaCl2, and 5 mM MgCl2 after centrifugation at 400× *g* for 8 min. The prepared antibody-coated beads according to the original blood volume were added afterwards. The cells were then incubated with the magnetic beads at 4 °C for 20 min.

### 2.4. CTC Enrichment and IsoFlux™

Immunomagnetic beads that target antigens expressed on the cell surface were used by IsoFlux^TM^ (Fluxion Biosciences Inc., South San Francisco, CA, USA). The beads were magnetic cores surrounded by a polymeric layer coated with a monoclonal human anti-mouse IgG antibody. CTCs were enriched in microfluidic devices in combination with primary mouse IgG antibodies. The CTCs and the beads were positively isolated from the sample using an immunomagnetic capture reagent, and the cells flowed through a microfluidic cartridge designed for cell isolation.

### 2.5. Sample Isolation and Collection

Beads containing CTCs were collected by following the enrichment protocol on an IsoFlux™ machine (Fluxion Biosciences Inc., South San Francisco, CA, USA). Isolated cells were then retrieved in 200 µL RPMI medium containing 1% fetal bovine serum (FBS), 5 mM MgCl2, and 1 mM CaCl2, and transferred to a low-retention microcentrifuge tube (Thermo Fisher Scientific Inc., Waltham, MA, USA). A cylinder magnet was used to pull down the cells bound to the beads towards the bottom of the tube while removing the supernatant. Cells were then fixed in 4% paraformaldehyde (PFA) and added onto glass slides, onto which a circle of the same size as the magnet was drawn using a water-repellent Dako pen. The glass slide was placed on top of the magnet when buffer was added or removed from the cells. This kept the beads and CTCs attached to the slides.

### 2.6. CTC Detection and Immunofluorescence

Isolated cells were mounted, fixed on slides, and blocked for 5 min in 10% normal donkey serum. The cells were then stained with a phycoerythrin-conjugated anti-CD45 [5B-1] antibody (1:200; MACS Miltenyi Biotec). After staining, cells were permeabilized using 0.2% Triton X-100 in PBS containing 2 mM EDTA and 0.5% BSA. FITC-conjugated anti-cytokeratin [CK3-6H5] antibody (1:10; MACS Miltenyi Biotec) was used for cancer cell staining. For cell nuclei, cells were incubated with 4′,6-diamidino-2-phenylindole (DAPI). The sample was mounted with Dako Faramount aqueous mounting medium. Images were captured using a fluorescence microscope (Axio Scan.Z1, Zeiss, Jena, Germany). CTCs were defined as nucleated (DAPI positive) cells with cytokeratin (CK)-positive and CD45-negative staining. 

### 2.7. Statistical Analysis

Unless stated otherwise, at least three independent experiments were performed. Statistical tests with their associated sample sizes are described in the results and figure legends. Unless stated otherwise, results for continuous variables are presented as mean ± standard deviation (SD), and statistical significance was set at *p* < 0.05. Data were analyzed using Prism version 8 (GraphPad Software Inc., San Diego, CA, USA).

## 3. Results

### 3.1. Patient Characteristics

The five patients enrolled in the control group all had clinical stage 2 disease. The mean ± SD age was 65.8 ± 5.3 years. Three (60%) patients were male and two (40%) were female. All of them had pathological stage 2 disease, with two (40%) being stage 2a, and three (60%) being stage 2b. According to the WHO histologic grading system, four (80%) were high grade and one (20%) was low grade. The detailed characteristics of the cohort of 33 MIBC patients are shown in Table 1. Mean ± SD age at diagnosis was 63.5 ± 6.6 years for these patients. Sixteen patients were male (48.5) and seventeen patients were female (51.5%). The patients’ clinical stages were as follows: stage 1, 0 patients (0%); stage 2, 25 patients (75.8%); stage 3, five patients (15.2%); and stage 4, three patients (9.1%). All patients underwent NAC for six courses and then underwent RC one month later. The pathological stages were as follows: stage 0, seven patients (21.2%); stage 1, 12 patients (36.4%); stage 2a, four patients (12.1%); stage 2b, one patient (3%); stage 3a, four patients (12.1%); stage 3b, two patients (6.1%); and stage 4a, three patients (9.1%). No pathologic stage 4b patient was found. Eight (24.2%) patients had low histologic grade and 25 (75.8%) had high histologic grade. The regimens of NAC were as follows: cisplatin-based, 26 patients (78.8%); carboplatin-based, 7 patients (21.2%).

### 3.2. Detection of Circulating UC Cells in Patients with MIBC

**Figure 1:**
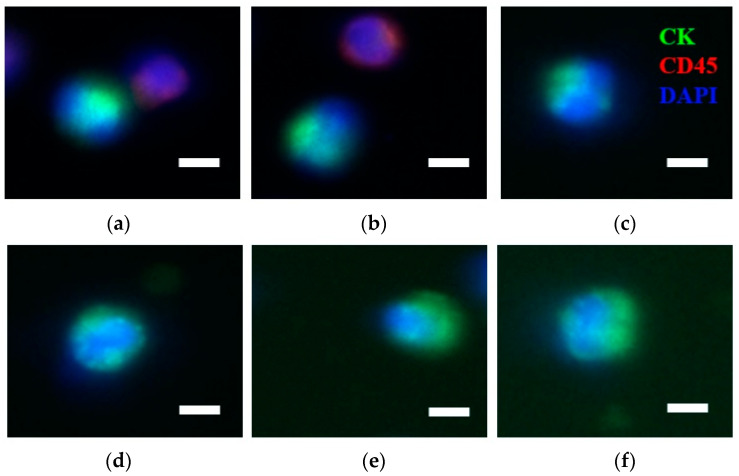
Circulating urothelial cancer cells are present in blood samples from patients. (**a**–**f**), Immunofluorescence staining of representative cells obtained from IsoFlux. Cancer cells fulfilled criteria for CTCs, including: nucleated (blue), CK-positive (green), and CD45-negative cells (non-red). Scale bar, 10 µm.

Immunofluorescence staining of the cells showed that CTCs were observed and isolated in all patients with MIBC (Figure 1a–f). Cancer cells were detected based on their morphology and their antigen expression. CTCs are nucleated (DAPI positive) cells with expression of cytokeratin (CK) but no CD45 expressed. In control patients, mean CTC numbers were 2.8 ± 0.8 per 7.5 mL before RC, and 0.8 ± 0.8 per 7.5 mL 2 weeks after RC. Surgical treatment with RC led to a significant decrease in the CTC number. Furthermore, CTCs were found in all patients with MIBC before treatment, after four cycles of NAC, and two weeks after RC. According to the change from clinical T stage to pathological T stage, patients with MIBC were categorized into the progressive disease (PD) and disease control (DC) groups. The PD group included patients with disease progression. The DC group included patients with a stable, partial response, and complete response disease. The relationship between the groups and the number of enriched CTCs is detailed in Table 2. A total of 19 patients (57.5%) were defined as the DC group and the enriched CTC numbers per 7.5 mL at baseline, after four cycles of NAC, and post-operation were as follows: 2.3 ± 0.7, 1.0 ± 0.6, and 0.3 ± 0.5. The other 14 patients (42.4%) were defined as the PD group and their enriched CTC numbers per 7.5 mL at baseline, after four cycles of NAC, and post-operation were as follows: 3.5 ± 1.6, 4.6 ± 2.1, and 1.2 ± 0.8.

### 3.3. Relationship between the Number of CTCs and Treatment Modality during Time Course

**Figure 2:**
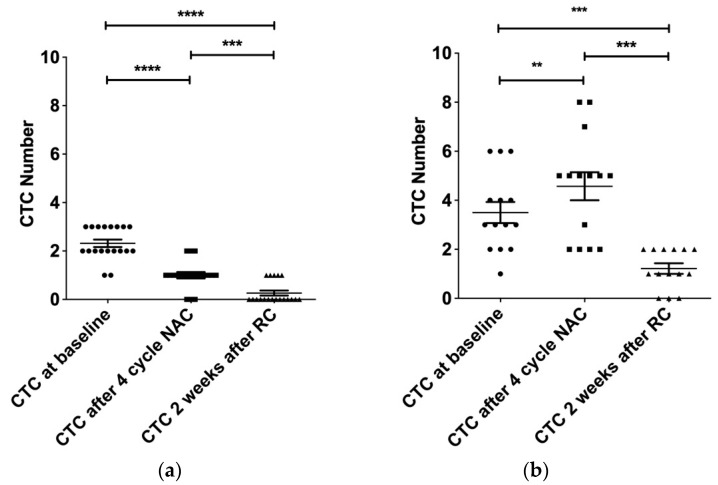
Time course analysis of CTC levels in bladder cancer. CTC levels were evaluated in 33 MIBC patients before starting treatment and after four cycles of NAC and two weeks after RC. Number of CTCs/7.5 mL is shown on the plots. (**a**) Disease control group; (**b**) Progressive disease group; Mann–Whitney test; ns, non-significant; ** *p* < 0.01; *** *p* < 0.001; **** *p* < 0.0001.

In the DC group, the number of CTCs decreased after four courses of NAC (Figure 2a, *p* < 0.0001). Furthermore, a decrease in CTCs was still noted after RC (Figure 2a, *p* < 0.001). In the PD group, the number of CTCs increased after four courses of NAC (Figure 2b, *p* < 0.01). However, the trend of CTC increase was reversed after RC (Figure 2b, *p* < 0.001). The counts of CTCs in patients with MIBC after four courses of NAC showed opposite results in the DC and PD groups. Compared with the four courses of NAC, surgical treatment with RC led to a decrease in the CTC number in these two groups.

### 3.4. Correlation of the CTC Values with the Tumor Stage

**Figure 3:**
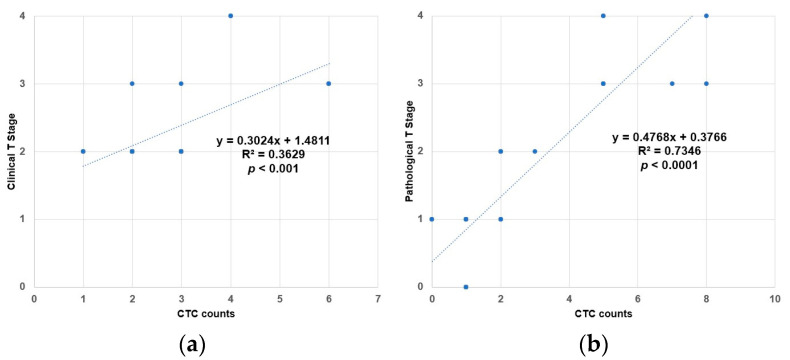
Correlation of CTC values with cancer T stage. (**a**) Univariate regression analysis between baseline CTC number/7.5 mL and clinical T stage; (**b**) Univariate regression analysis between CTC number/7.5 mL after four cycles of NAC and post-operative pathological T stage.

Univariate regression analysis was performed to explore the association between CTCs and tumor stage in patients with MIBC over time. The relationship between baseline CTC counts and initial clinical cancer stage is shown, and a statistically significant linear correlation was found (Figure 3a, *p* < 0.001). After four courses of NAC, CTC samples were collected separately. The relationship between pathological tumor stage and CTC count was revealed, and a statistically significant linear correlation was also noted (Figure 3b, *p* < 0.0001).

## 4. Discussion

Based on the latest guidelines, the standard systemic therapy for patients with MIBC is platinum-based NAC, which consists of cisplatin, methotrexate, doxorubicin, and vinblastine followed by RC [2]. RC with bilateral pelvic lymphadenectomy is the therapeutic basis for MIBC. Surgical intervention can provide definite pathological T stage, lymph node invasion status, and prolong cancer-specific and overall survival [2]. Several possible advantages of application for NAC in patients with MIBC before RC are mentioned in the literature. First, the increasing opportunity to control the micro-metastatic disease while the tumor burden is not large [2]. Second, administration of NAC before RC provides effective drug delivery to peri-vesical tissues, lymphatic vessels, and regional lymph nodes under original anatomic structure [19]. Third, better tolerance to standard platinum-based regimen while the patient’s performance status is optimal before a major operation. The most important advantage is the overall survival benefit for patients with MIBC. Compared with RC alone, addition of NAC for patients with MIBC not only increases by 5–10% the absolute overall survival benefit, but it also decreases 16–33% of the relative risk of death [6,7,20]. On the contrary, the neoadjuvant setting might impair general health condition due to chemo-toxicity of hematologic or gastrointestinal side effects and result in increasing perioperative morbidity or postoperative complication [19]. Regarding these concerns, several studies have revealed no significant difference in blood transfusion, perioperative mortality, postoperative complication, or readmission rate in NAC-treated patients followed by RC. NAC seems to be a harmless approach to patients with MIBC [21,22]. Another disadvantage is that NAC might delay surgical intervention, especially in non-responders. Not all patients with MIBC show an excellent response to NAC. Pathological complete response was only observed in 24.3–25.7% after neoadjuvant cisplatin-based chemotherapy [3]. To date, no credible tool has been applied to predict therapeutic responses to NAC. Distinguishing non-responders to NAC to prevent delayed surgical intervention is currently challenging. According to our best knowledge about predictors for therapeutic response of NAC, no previous studies discuss the relationship between the quantitative analysis of CTCs and the efficacy of NAC for tumor down-staging. Thirty-three MIBC patients who received NAC and RC were enrolled in this study. Our research shows that CTC numbers after four cycles of NAC in patients with MIBC are highly correlated with postoperative pathological tumor stage. This result revealed the predictive potential of CTCs in MIBC patients undergoing NAC therapy. Furthermore, the dynamic change in CTC counts between baseline and four cycles of NAC showed significant differences between the DC and PD groups. Dynamic changes in CTC counts after four cycles of NAC seem to be closely correlated with changes in postoperative tumor stage. Thus, CTCs can be a good predictor of NAC therapeutic responses. In the future, patients who are not going to have a good response to NAC should receive RC earlier.

In 1869, CTCs were observed in the blood for the first time from a man with metastatic malignant tumor by the Australian physician Thomas Ashworth [23]. CTCs are cells shed from primary tumors or metastatic sites which subsequently enter the bloodstream, travel to distant organs, and lead to the formation of secondary metastatic tumors. In patients with metastatic cancer disease, CTCs can be detected in frequencies around 1–10 CTC per mL of whole blood [24]. As such, the isolation, identification, and characterization of CTCs require highly specific and sensitive technologies. Despite significant improvements in emerging technology over the past decades, it still has not been possible to achieve a complete satisfaction due to tumor heterogeneity and cancer dynamics. The separation of CTCs from the blood of cancer patients holds great potential as a minimally invasive approach, but it is undoubtedly challenging. Platforms and methods for the enrichment of CTCs vary significantly concerning the underlying technology, ranging from physical property-based platforms, functional assays, and antibody-based approaches. CTCs are able to initiate metastasis and bear valuable information for cancer diagnosis and disease monitoring. Recent publications have already demonstrated the predictive potential of CTC numbers [13,17,23,25,26,27,28,29,30,31,32,33,34]. Since metastatic and primary tumors continuously release CTC, the enumeration of CTCs can provide valuable information in the clinic, such as serving as a biomarker for screening and early detection, real-time monitoring of therapy response, and prediction of prognosis [35,36,37,38,39]. Furthermore, when no biopsy material can be collected or when serial biopsies of a tumor are practically not possible, CTCs provide a non-invasive source for tumor material. This ‘liquid biopsy’ is particularly crucial for patients. However, the biological characterization and molecular analysis of CTCs have stayed at a somewhat experimental stage [40,41]. Molecular analyses of CTCs have shown a potential to be biomarkers that can be used for monitoring treatment response [35,36]. Changes in the number of CTCs can be detected and used for assessment of disease response to therapy in different cancer types. In metastatic breast cancer, data from 1944 eligible patients from 17 international medical centers showed the independent predictive value of CTC quantification on overall survival and progression-free survival. Moreover, CTC counts improved the prognosis of disease when added to full clinical and pathological predictive models, whereas other tumor markers usually used in clinics do not [37]. The COU-AA-301 trial is a randomized, double-blind, multinational phase III trial of abiraterone acetate plus prednisone versus prednisone alone in metastatic castration-resistant prostate cancer (CRPC) patients previously treated with docetaxel. The results revealed that individual patient CTC number was related to survival, whereas changes in serum PSA levels, which are currently used in clinics, were not relevant [38]. Conversion of baseline unfavorable CTCs (>3 CTCs/7.5 mL) to favorable at 3 to 5 weeks in colorectal cancer was shown to be correlated with significantly longer OS and PFS compared with patients with only unfavorable CTCs [39]. Treatment of cancer is usually complicated by the nature of the disease, tumor heterogeneity, genes and pathways related to the organs, and drug resistance. Characterization of CTCs can provide information for predicting treatment response, especially for identifying treatment targets. CTCs with specific expression can further offer a new therapeutic selection for treatment in several cancer types. In urothelial carcinoma, our group demonstrated that ongoing changes in CTCs with expression of PD-L1 impact the oncological response to anti-PD-L1 treatment. A decrease in these specific CTCs is a suitable predictor for prognosis and therapeutic response to PD-L1 inhibition therapy [13]. Additionally, in renal cell carcinoma, we also showed that the existence of CTCs with expression of PD-L1 before PD-1 blockade therapy could be a predictive factor for disease prognosis and that the dynamic changes in CTC counts may be used to monitor treatment response. Our previous study confirmed that in clear cell renal cell carcinoma patients’ blood samples, PD-L1-positive CTC detection can be used as a clinical biomarker for real-time assessment [33]. These findings suggest CTCs can be a ‘liquid biopsy’ able to predict which patients will benefit from PD-1 inhibition therapy, a currently promising therapeutic approach in several cancers.

In our study, we found that CTC numbers after RC showed a significant decrease compared with those after four cycles of NAC in both the DC and PD groups. This trend was compatible with the dynamic changes in CTC counts after four cycles of NAC in the DC group. The decrease in CTC number/7.5 mL after RC may reflect the surgical effect in patients with MIBC in the current study. Clinical staging of bladder cancer is used to guide treatment plans and to estimate tumor prognosis. In our study, CTCs in patients with MIBC at baseline were significantly correlated with the initial clinical T stage. This finding can provide clues regarding disease severity before invasive procedures are performed in patients with bladder cancer. In previous publications, CTCs were also used to investigate outcomes in UC patients who underwent RC. Soave et al. enrolled 185 patients in their study. Compared with patients with CTCs, those without CTCs received adjuvant chemotherapy less often. Multivariable analysis revealed that the existence of CTC was an independent prognosticator of disease progression and cancer-specific survival [42]. The study reminded us that the status of CTC might be applied to judge the necessity of adjuvant chemotherapy in patients with UC who underwent RC even without adverse features, such as extravesical disease.

Several clinical, molecular, histological, and imaging factors have been evaluated for predicting the response of NAC [8]. In bladder cancer, tobacco smoking is considered to be the most common risk factor, and the strength and course of smoking also affect the severity of bladder cancer [43]. However, the relationship between the response to NAC in patients with MIBC and smoking status remains unclear. Boeri et al. enrolled 201 patients with MIBC and categorized smoking status as never smokers (28.9%), former smokers (43.3%), and current smokers (27.9%) [44]. Former smokers and current smokers were significantly correlated with a poorer response to NAC. Moreover, current smoking was also correlated with a risk increase of disease recurrence after RC. Magnetic resonance imaging (MRI) has also been reported to have a role in predicting treatment response in patients with MIBC undergoing induction chemoradiation therapy. The apparent diffusion coefficient (ADC) values of diffusion-weighted MRI showed a negative correlation with pathologic complete response [45]. Shimaa et al. also demonstrated a similar result regarding the utility of multiparametric MRI to predict the treatment response of NAC for patients with MIBC. The pathologic complete response in cystectomy specimens after NAC was negatively correlated with ADC values on diffusion-weighted MRI [46]. These two studies showed the potential ability of multiparametric MRI to predict response to neoadjuvant therapy in patients with MIBC. MIBC molecular subtyping has also been investigated to predict neoadjuvant therapeutic response [47]. Seiler et al. reported the identification of molecular subtypes (basal, claudin-low, luminal, and luminal-infiltrated) using a single-sample genomic subtyping classifier to predict the response to NAC in MIBC patients [48]. Due to the highly proliferative character of basal tumors, this subtype achieved the most extended overall survival for NAC plus RC compared with RC alone. Little or no benefit was found in patients with subtypes of luminal tumors and claudin-low tumors with or without NAC. Another interesting finding in patients with a subtype of luminal-infiltrated tumors seems to be the benefit of immunotherapy with atezolizumab. This study shows the potential of the molecular subtype of MIBC in choosing a suitable patient for NAC. Nevertheless, molecular subtype is not able to be a treatment response predictor.

Taken together, this study demonstrates that changes in CTC counts can contribute to the early evaluation of therapeutic effects. Compared with the above-mentioned parameters for predicting the response to NAC in patients with MIBC, our study showed a significant quantitative relationship between CTC counts and response to NAC. To our knowledge, our finding is the first study to discuss the predictive potential of CTCs in response to NAC in MIBC patients. However, our study has some limitations. The relatively small sample size is the first limitation of this study. Most patients with MIBC refuse RC after NAC because of fear of complications of radical surgery. The second limitation is the NAC regimen. Not all patients with MIBC received the same regimen of NAC because of impairment of renal function. Cisplatin-based NAC is the standard therapeutic choice for patients with MIBC. Different regimens of NAC might influence the number of CTCs and therapeutic response by varying degrees. Third, the short follow-up time makes the demonstration of overall survival impossible in this study. Last, our epithelial markers for antibody-based CTC enrichment platform certainly misses out on CTCs that have undergone epithelial to mesenchymal transition (EMT). Limited availability of specific CTC surface markers as well as a varying degree of marker specificity across cancer types provide a challenging environment for establishing a globally useful CTC isolation technique.

## 5. Conclusions

The standard treatment for MIBC patients is effective NAC, followed by RC. The identification of non-responders to NAC to decrease chemotherapy-related side effects and to prevent delayed RC is crucial in clinical practice. However, a validated parameter for predicting response to NAC is still lacking. Our study revealed that dynamic changes in CTC counts after four cycles of NAC in patients with MIBC are highly correlated with NAC response. Furthermore, CTC counts in 7.5 mL after four NAC cycles were highly correlated with postoperative pathological T stage. By detecting CTCs in patients with MIBC, early RC could be arranged in non-responders to NAC. Further studies with long-term follow-up and larger cohorts are necessary for the application of CTCs to predict the therapeutic response to NAC and possible non-invasive monitoring of disease progression in patients with MIBC.

## Figures and Tables

**Table 1:** Characteristics of enrolled patients.

Age (Mean ± SD) 63.5 ± 6.6	No. of Patients 33	Percentage 100
Gender		
Male	16	48.5
Female	17	51.5
Grade		
Low	8	24.2
High	25	75.8
Clinical T Stage		
1	0	0
2	25	75.8
3	5	15.2
4	3	9.1
Pathological T Stage		0
0	7	21.2
1	12	36.4
2a	4	12.1
2b	1	3
3a	4	12.1
3b	2	6.1
4a	3	9.1
4b	0	0
Chemotherapy regimen		
Cisplatin	26	78.8
Carboplatin	7	21.2

No. number, SD standard deviation.

**Table 2:** Enriched CTC numbers.

No. of Patients	Baseline	CTC Numbers/7.5 mL after Four Cycles of Neoadjuvant Chemotherapy (Mean ± SD)	2 Weeks after Radical Cystectomy
DC group: 19	2.3 ± 0.7	1.0 ± 0.6	0.3 ± 0.5
PD group: 14	3.5 ± 1.6	4.6 ± 2.1	1.2 ± 0.8

CTC circulating tumor cells, DC disease control, No. number, PD progressive disease, SD standard deviation.

## Data Availability

Not applicable.
